# Morphology and composition of the ventral neck muscles in individuals with chronic whiplash related disorders compared to matched healthy controls: a cross-sectional case–control study

**DOI:** 10.1186/s12891-022-05811-x

**Published:** 2022-09-16

**Authors:** Anneli Peolsson, Anette Karlsson, Gunnel Peterson, Hanna Borén, Peter Zsigmond, James M. Elliott, Olof Dahlqvist Leinhard

**Affiliations:** 1 Occupational and Environmental Medicine Center, Department of Health, Medicine and Caring Sciences, Unit of Clinical Medicine, Linköping University, Linköping, Sweden; 2 Department of Health, Medicine & Caring Sciences, Unit of Physiotherapy, Linköping University, Linköping, Sweden; 3 Center for Medical Image Science and Visualization (CMIV), Linköping University, Linköping, Sweden; 4 Centre for Clinical Research Sörmland, Uppsala University, Eskilstuna, Sweden; 5 Department of Neurosurgery and Department of Biomedical and Clinical Sciences, Linköping University, Linköping, Sweden; 6 The University of Sydney and the Northern Sydney Local Health District, The Kolling Institute, St. Leonards, NSW Sydney, Australia; 7 Department of Health, Medicine & Caring Sciences, Linköping University, Linköping, Sweden

**Keywords:** Spine, Neck muscles, Whiplash injury, Magnetic resonance imaging, Area, Fat

## Abstract

**Background:**

**Objective:**

Studies of cross-sectional area (CSA) (morphology) and muscle fat infiltration (MFI) (composition) in ventral neck muscles is scarce in patients with chronic whiplash associated disorders (WAD), especially for men and those with severe WAD compared with matched healthy controls. The aim was to compare CSA and MFI of sternocleidomastoid (SCM), longus capitis (LCA) and longus colli (LCO) in patients with chronic right-sided dominant moderate (Neck Disability Index: NDI < 40) or severe WAD (NDI ≥ 40), compared to age- and sex-matched healthy controls.

**Methods:**

Cross-sectional case–control study with blinded investigators. Thirty-one patients with chronic WAD (17 women and 14 men, mean age 40 years) (SD 12.6, range 20–62)) and 31 age- and sex-matched healthy controls underwent magnetic resonance imaging of ventral neck muscles segmental level C4.

**Results:**

Unique to the severe group was a larger magnitude of MFI in right SCM (*p* = 0.02) compared with healthy controls. There was no significant difference between the groups with regards to the other muscles and measures.

**Conclusions:**

Individuals with severe right-sided dominant WAD have a higher MFI in the right SCM compared to healthy controls. No other differences were found between the groups. The present study indicates that there are changes in the composition of muscles on the side of greatest pain.

## Introduction

Whiplash injury from a motor vehicle collision (MVC) exposes the cervical spine to rapid mechanical forces [1–3], often exceeding the thresholds for cervical muscle strain injury [4]. MVC related whiplash is common with an annual incidence of 0.6% of inhabitants in the Western world [1]. Roughly half of those affected should expect to recovery spontaneously but the other half may transition from acute to chronic whiplash associated disorders (WAD), negatively impacting daily and working life [5–8]. WAD symptoms such as neck pain, radiculopathy, neck-specific disability, headache and dizziness may be related to structural injury [9–12] where the function of muscles traversing the cervical spine is impaired [13]. Neck-specific exercise aimed at restoring impaired muscular activation [7, 12, 14, 15] has shown promising results in chronic WAD [16–18] indicating the importance of a well-functioning neck muscle “corset”.

Reports of altered cross-sectional area (CSA) or relative CSA (rCSA) of select, mainly dorsal, muscles in WAD compared with healthy controls has been inconsistent [10, 19–23]. Reduced CSA may be a consequence of atrophy, and higher muscle fat content (fat vs contractile muscle components) the consequence of pseudohypertrophy. Either way, both result in lesser contractile muscular content and this may be a feature of the reported impairments in roughly half of those transitioning to chronic WAD. It is acknowledged that the evidence for early risk-based interventions is limited [8, 24] and there is a ‘global messaging’ to reduce unnecessary imaging. However, there remains a need to improve our understanding of biological based risk factors (e.g., quality and quantity of the muscles traversing the cervical spine [25] in individuals with acute injury). Such knowledge may provide foundation for exploring and establishing an improved diagnostic, prognostic, and therapeutics landscape for patients where few options exist [10, 20, 25–28].

Superficial ventral neck muscle sternocleidomastoid (SCM), deep ventral longus capitis (LCA) and longus colli (LCO), CSA and muscle fat infiltration (MFI) have not been widely studied in patients with neck pain or cervicogenic headache and without consistent findings [20, 23, 25, 29–31]. Studies performed on ventral neck muscles have been without group blinded assessor, and except for CSA measurements in the study by Ulbrich et al. [31] in acute mild (grade 1) and moderate (grade 2) WAD, were without age- and sex-matched healthy controls. Only in a few studies were individuals with chronic WAD included [20, 23, 25]. Men and those with severe WAD with both musculoskeletal and neurological findings (WAD grade 3) have been excluded and sometimes LCO and LCA have been combined in analysis. An important knowledge gap is thereby present in the understanding of WAD pathophysiology, especially as ventral muscles have been shown to be a key factor in rehabilitation [12, 14].

The aim of the present study was to compare CSA and MFI for select ventral muscles (Sternocleidomastoid (SCM), Longus Colli and Capitis (LCO and LCA) muscles in right-handed men and women with chronic right-sided dominant moderate (Neck Disability Index: NDI [32] < 40%) or severe WAD (NDI ≥ 40%) [10, 26, 28] compared with right- handed age- and sex-matched healthy individuals.

The hypothesis of the study was that MFI would be higher in individuals with WAD compared with healthy controls, and that those with severe WAD would have higher MFI compared to those with moderate WAD. Regarding CSA, differences between groups may be expected, but based on the literature, we did not have a definite direction as hypothesis.

## Methods

This is a cross-sectional case–control study with investigators blinded for group belonging when performing the magnetic resonance imaging (MRI) muscle segmentation. Data was collected at an University hospital in the south of Sweden during 2012 and 2013, later on segmented and analysed.

### Ethical statement

Human experimentation was approved by the local regional ethics committee at Linköping University, Sweden before data collection started (Dnr 2011/262–32) and conforms to Helsinki Declaration. All experiments were performed in accordance with relevant Swedish guidelines and regulations.

### Participants

Inclusion criteria for the WAD group were persistent neck problems ≥ 6 months but ≤ 3 years after a whiplash injury [33]; age 18–63 years; neck pain > 20 mm on a Visual Analogue Scale; and > 20% on the NDI [16, 34, 35].

Exclusion criteria were neck pain that caused a > 1 month absence from work in the year prior to the WAD trauma, known or suspected serious physical pathology, surgery on the cervical spine, signs of traumatic brain injury at the time of whiplash injury, diseases or other injuries that might prevent full participation in the study, generalised or more dominant pain elsewhere in the body and insufficient knowledge of the Swedish language (inability to answer the questionnaires and to understand instructions) [16, 34, 35]. Additional exclusion criteria in the present study were contraindications for MRI such as shunt, pacemaker, metal in the body, claustrophobia, body mass index (BMI) > 35 (due to the space within the magnet) and pregnancy [10, 26, 28].

A convenience sample of age- and sex-matched controls that felt healthy (*n* = 31) were consecutively recruited for each of the included patients (Table 1). The healthy (right-handed) controls presented without present or recurrent neck pain, never received a trauma against the neck or head and never received treatment for neck pain. The control group did not differ regarding height, weight, or BMI in relation to the patients [10, 26, 28].

**Table 1:** Demographic table for healthy controls (controls) and patients with chronic moderate (Neck Disability Index: NDI < 40) or severe (NDI ≥ 40) whiplash associated disorders (WAD)

	**Controls**	**WAD < 40**	**WAD ≥ 40**	**Significance**
**Total number**	31	20	11	
**Female/Male**	17/14	10/10	7/4	*P* = 0.77*
**Age** Years (range)	41.5 (22—61)	39.2 (20—62)	45.7 (33—58)	*p* = 0.26**
**Duration** Months (range)	N/A	20 (7—36)	14,5 (6—32)	*p* = 0.11***
**NDI** (mean (range))	N/A	27.3 (10—38)	51.3 (40—68)	*p* < 0.0001***
**VAS** (mean (range))	N/A	30.2 (3—70)	53.5 (30—75)	*p* = 0.005***

*VAS* Visual analoge scale

^* ^Chi^2-test used

^** ^ANOVA

^*** ^t-test 2-sided

### MRI analyses

MRI images were acquired with a Philips Ingenia 3.0 T scanner (Royal Philips, Amsterdam, the Netherlands), using the built-in phased-array posterior coil, a 32-channel head coil and an anterior flexible coil placed adjacent to the head coil. The participants were positioned supine for imaging. A three-dimensional gradient-echo sequence was used with out-of-phase and in-phase echo times of 3.66 ms and 7.24 ms, respectively. The echo times were chosen to enable high resolution. The repetition time was 10 ms and the flip angle was 10°, with a total acquisition time of 9.07 min. The images included cervical segmental level C4 and were angled so that the in-plane images were parallel to the cervical segments. The acquired image resolution was 0.75 × 0.75 × 0.75 mm^3^. Phase-sensitive reconstruction was used to acquire fat- and water-separated images [10, 26]. With dual-echo mDixon imaging it is not possible to correct for T2 effects based on the information in the images. Therefore, a literature value for T2 relaxation of 23.9 ms was used for both fat and water to perform the T2 correction in this study [10, 20].

### Muscle segmentation

MRI muscle segmentation (Analyze V 11.0, AnalyzeDirect, Inc, Overland Park, USA) at C4 segmental level [20] were performed in random order by an investigator, a musculoskeletal physiotherapist blinded for group belonging (HB in discussion with AKK, AP and ODL) with good to excellent test–retest reliability (two-way random absolute agreement single measure intra-class correlation coefficient (ICC)) for muscle CSA (ICC 0.93–0.98) and MFI (ICC 0.70–0.99). The test–retest analysis was randomly performed of 20 randomly selected duplicates (HB and JME were unaware of the duplicated imaging). A second experienced investigator (JME) approved all segmentations (musculoskeletal physiotherapist and Professor with more than 15-years’ experience of MRI neck muscle segmentation). The investigators (HB and JME) were independent and not involved in study design or recruitment of participants.

**Fig. 1:**
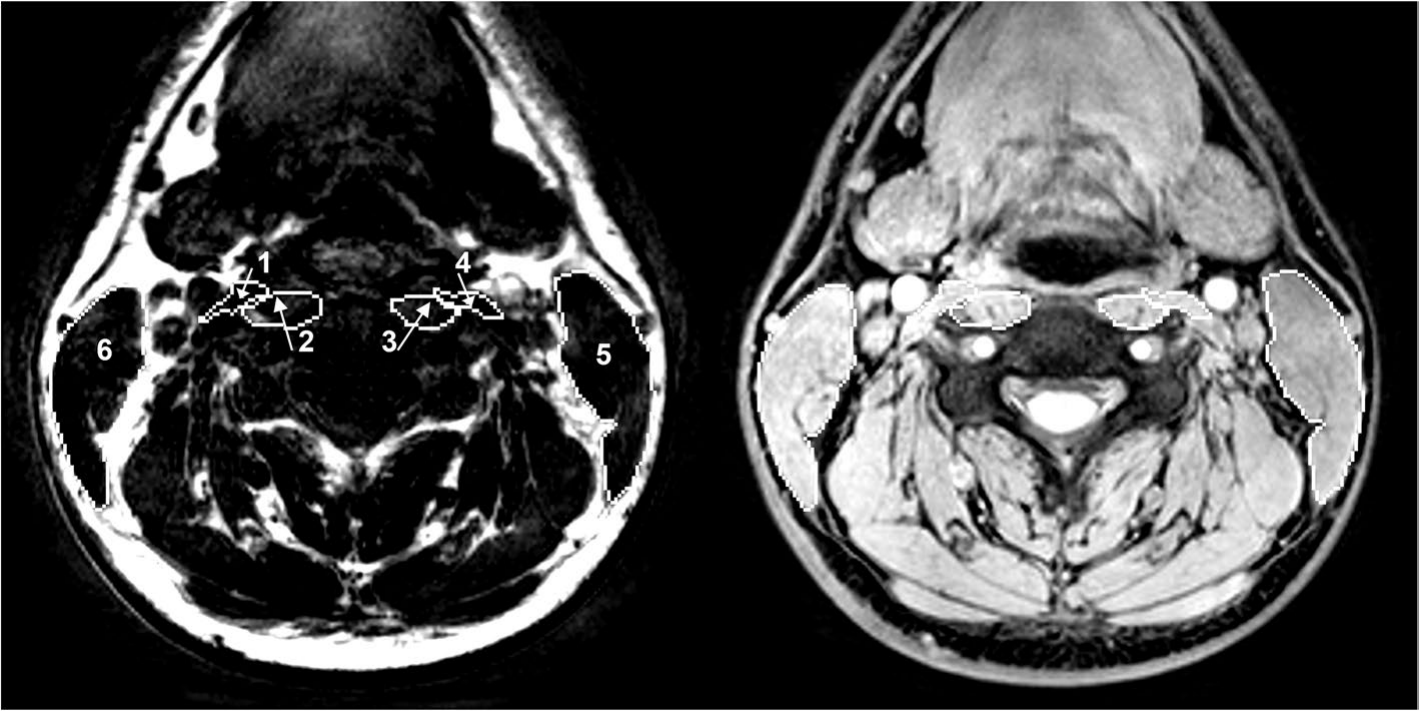
Region of interest (ROI) of the longus colli (2, 3), longus capitis (1, 4) and sternocleidomastoid (5, 6). To the left a fat imaging and to the right a water imaging at C4 segmental level

Manual muscle segmentation with identification of region of interest (ROI) of SCM, LCA and LCO (Fig. 1) was performed with axial fat- and water imaging, where the ROIs were drawn on the water imaging with visual support from the fat imaging and checked for accuracy. The water imaging, fat imaging and the mixed water-fat imaging were checked by visual inspection a final time before the second investigator rechecked and approved all ROIs. The ROIs were downloaded into MATLAB 2013b (Math Works, Inc, MA USA). The MFI were calculated as the fat within the ROI divided by the total sum of all water and fat within the muscle. The CSA was calculated in square millimetres (mm^2^) [10].

### Statistical analysis

The WAD group was divided in a moderate (NDI < 40%, *n* = 20) and a severe group (NDI ≥ 40%, *n* = 11) [20, 26, 28]. The Kruskal–Wallis one way analysis of variance was used for analyses between the three groups; when a significant difference (*p* ≤ 0.05) was identified a Mann–Whitney U-test was used as a post-hoc test.

Wilcoxon signed-rank test was used to analyse differences between right and left SCM in interpretation of the findings. IBM SPSS Statistics for Windows version 26 was used for statistical analyses. Statistical analysis was performed by a university statistician (not involved in study design or data collection) in dialogue with AK and AP.

## Results

Thirty-one people (17 women and 14 men, with a mean age of 40 years (SD 12.6)) with chronic WAD (≥ 6 month duration) with a mean symptom duration of 20 months (SD 9) who were right-handed and with right-sided dominant symptoms (right-side only pain or bilateral neck pain worse on the right side) and signs of WAD grade 2 (clinical musculoskeletal findings emanating from the neck) and 3 (as grade 2 but with additional neurological findings) [33], were consecutively recruited at baseline from an ongoing randomised controlled study [16, 34, 35] after oral and written informed consent (Table 1). Right-handed patients with dominant right-sided problems were chosen so that handedness would not imply a bias in the analysis.

Except for MFI of the right SCM (*p* = 0.03) with the significant differences between severe WAD group and control group (*p* = 0.02), there were no significant differences between the three groups, neither for CSA (Table 2) or MFI (Table 3).

**Table 2:** Between groups comparison of cross-sectional area (mm^2^) of ventral neck muscles (longus colli = LCO, longus capitis = LCA, sternocleidomastoid = SCM)

Muscle	LCO left	LCO right	LCA left	LCA right	SCM left	SCM right
WAD < 40*						
Median (mm^2^)	61.6	71.2	55.4	57.4	400.5	426.9
Range (mm^2^)	73.7	52.3	32.6	38.8	248.6	249.2
Number	20	20	20	20	14	15
(Drop-out*)	(0)	(0)	(0)	(0)	(6)	(5)
WAD ≥ 40*						
Median (mm^2^)	52.9	59.6	43.9	47.8	323.2	354.4
Range (mm^2^)	51.2	43.9	33.2	3.9	284.6	313.3
Number	9	9	9	9	8	9
(Drop-out*)	(2)	(2)	(2)	(2)	(3)	(2)
Controls*						
Median (mm^2^)	63.0	65.3	50.3	54.6	403.9	404.4
Range (mm^2^)	50.1	56.3	68.6	56.3	363.4	333.6
Number	28	28	28	28	25	27
(Drop-out)	(3)	(3)	(3)	(3)	(6)	(4)
*p* =	0.22	0.09	0.11	0.23	0.22	0.26

*WAD Whiplash Associated Disorders, NDI* Neck Disability Index

^*^ moderate WAD (NDI < *40), severe WAD (NDI* ≥ *40) and age- and sex-matched healthy controls. Drop-out is unclear imaging, not possible to analyse*

**Table 3:** Between groups comparison of muscle fat infiltration of ventral neck muscles (longus colli = LCO, longus capitis (LCA) and sternocleidomastoid = SCM)

Muscle	LCO left	LCO right	LCA left	LCA right	SCM left	SCM right
WAD < 40*						
Median (%)	-0.005	-0.006	-0.04	0.009	0.059	0.041
Range (%)	0.25	0.32	0.22	0.20	0.32	0.30
Number	20	20	20	20	14	15
Drop-out	(0)	0	(0)	(0)	(6)	(5)
WAD ≥ 40*						
Median (%)	0.026	0.054	0.011	0.023	0.110	0.104
Range (%)	0.30	0.36	0.33	0.11	0.18	0.21
Number	9	9	9	9	8	9
Drop-out	(2)	(2)	(2)	(2)	(3)	(2)
Controls*						
Median (%)	0.019	0.019	0.007	-0,017	0.062	0.019*
Range (%)	0.32	0.41	0.31	0.34	0.25	0.43
Number	28	28	28	28	25	27
Drop-out	(3)	(3)	(3)	(3)	(6)	(4)
*p* =	0.39	0.75	0.71	0.35	0.25	0.03

*WAD*   Whiplash Associated Disorders*, NDI* Neck Disability Index

^*^WAD moderate (NDI < 40), severe WAD (NDI ≥ 40) and age- and sex-matched healthy controls

Control vs. severe WAD *p* = 0.02, control vs. mild WAD *p* = 0.12, mild WAD vs. severe WAD *p* = 0.10 (Mann–Whitney U-test)

SCM left vs. right (Wilcoxon signed rank test) *p* = 0.01 for healthy controls

Drop-out = unclear imaging, impossible to analyse

## Discussion

Except for significant higher MFI in the severe WAD group (about 550% higher) in the right SCM compared with the healthy group, there were no significant differences in muscle volume or MFI between the three groups in the present study. In the moderate WAD group there was about 220% higher MFI in right SCM compared with the healthy group, showing the same trend as in the severe group but without significant differences. The result between groups for left SCM, LCA and LCO did not differ if moderate and severe WAD groups were combined against the healthy group, with no significant differences between groups (*p* = 0.11–0.90), and was independent of parametric or non-parametric statistics, showing a stability in the results. There were no significant differences between the three groups regarding CSA. The hypothesis was thereby only met regarding significant higher MFI in the severe WAD group and only at the (most) affected right side. CSA and MFI for SCM has been reported to be higher, lower or without differences from healthy [20, 23, 27, 30, 31] and LCO and LCA to be increased or without differences from healthy [20, 30]. In studies of individuals with chronic WAD (dominant side of pain and handedness not defined in moderate WAD and healthy controls unmatched), increased MFI in SCM against healthy controls were reported by Van Looveren et al. [23] and Elliott et al. [20]. However, the results by Elliott et al. [20] was contradictory to the present study, with the highest MFI found in the deep ventral neck muscles and not in the SCM. When the fat signal was removed from the MRI imaging in the study by Elliott et al. [25] the results regarding muscle size of SCM was inconsistent between healthy and controls. Differences in findings among the studies may be caused by different aims, study populations, scanning techniques, different segmental levels measured, different measurements of CSA, MFI and combining muscles [9, 39].

In the present study, *higher* MFI was found in the right SCM in the WAD group, but not the left side. This may be explained by how all in the WAD group had dominant right-sided problems with right as their worse side. SCM distributes high amounts of load [4, 36], exceeding the injury threshold, during high acceleration and strain from rear and lateral impacts [4, 37]. It remains plausible findings of higher MFI on the painful side could be the result of an injury mechanism that exceeded the individual’s strain threshold. Cagnie et al. [29] found *lower* MFI in the right SCM compared with the left side in asymptomatic individuals. This is the opposite findings compared with the WAD group, this even though the WAD group were right-handed, which strengthens the group differences.

During the last decade a number of theories underlying greater changes in the deep muscles feature in those with acute and chronic WAD. Despite the lack of understanding the precise mechanisms underlying these changes, findings from the basic sciences point to specific molecular processes. For example, the micro ribonucleic acid (RNA) let-7i-5p, which participants in the recruitment and function of brite adipocytes [38], was shown to mediate the relationship between MFI and neck disability after a whiplash injury, giving a new piece in the complicated puzzle [39]. There remains inconclusive evidence if the muscles changes are local to the site of injury (e.g., cervical spine) or extend to the distal muscles, suggesting a more systemic consequence [40]. There is emerging evidence that changes in the composition of distal muscles may reflect a more severe injury involving the spinal cord; changes that are radiologically occult when using conventional imaging techniques. While the number of participants damaging their spinal cord in a simple MVC is likely low, its occurrence is not impossible [40].

### Study limitations

Previous work [10], involving the same cohort, and others [41] has reported significant degeneration with increased MFI in the deep dorsal multifidus muscle. This has also been shown in the deep ventral LCO and LCA muscles in other cohorts [20, 30]. We were thus surprised by the lack of group differences in the deeper ventral muscles in this cohort. However, lower MFI has been reported in ventral compared with dorsal neck muscles in asymptomatic [42] and symptomatic populations [20, 43]. The muscle fibre composition differs between deep dorsal and ventral neck muscles, with more slow oxidative type 1 fibres in dorsal multifidus [44] that may be prone to transform into fast type IIB fibres in WAD followed by increased MFI [20, 27, 44]. However, from the present study we cannot exclude that such findings would be present in LCO and LCA in other cervical segments not investigated or in regions of the muscles as reported for the multifidus muscle [26]. Another drawback is that the present study may be underpowered and future larger studies are needed. Manual segmentation is extremely time consuming and MRI investigations rather expensive, so advanced automated segmentation and future analyses with help from artificial intelligence may be helpful in future research [45, 46]. No age- or gender-related differences in measured parameters were studied. The sample size was too small for subgrouping on age and gender and this was not the aim of the study.

### Study strengths

The strength of the present study was that all patients had a WAD verified in a clinical examination, it matched age- and sex-specific healthy controls, with excellent test–retest reliability of MRI analyses, and analyses of each muscle separately and for both right and left side, and investigators blinded for group belonging. Both investigators were independent and not involved in study design or data collection. MFI was analysed additional to CSA measurements. Furthermore, in the present study both sexes and even those with severe WAD were included. Another advantage was that all patients reported right-sided problems. This made the present study unique, contributing to the scientific knowledge of those with WAD.

## Conclusions

One can conclude that individuals with severe right-sided dominant WAD (NDI ≥ 40%) have higher MFI in the right SCM compared with healthy individuals, but that there were no significant differences in CSA or in LCO and LCA. The present study indicates that there are compositional changes in the SCM on the painful side and that it may be important to exercise the SCM muscle in individuals with persistent WAD. However, this needs to be further evaluated in longitudinal intervention studies. Further studies are also needed to investigate the functional importance of MFI in ventral neck muscles. The study result may be generalised to individuals with chronic WAD grade 2 and 3, we believe even to those left-handed with left sided dominant pain.

## Data Availability

Data can be available upon reasonable request to Anneli Peolsson (Anneli.Peolsson@liu.se) and after ethical permission.

## Abbreviations

BMI: Body mass index
CSA: Cross-sectional area
LCA: Longus capitis muscle
LCO: Longus colli muscle
MFI: Muscle fat infiltration
MRI: Magnetic resonance imaging
MVC: Motor vehicle collision
NDI: Neck disability index
rCSA: Relative CSA
RNA: Micro ribonucleic acid
ROI: Region of interest
SCM: Sternocleidomastoid muscle
VAS: Visual analogue scale
WAD: Chronic whiplash associated disorders

## References

[1] Holm LW, Carroll LJ, Cassidy JD, Hogg-Johnson S, Côté P, Guzman J (2008). The burden and determinants of neck pain in whiplash-associated disorders after traffic collisions: results of the bone and joint decade 2000–2010 task force on neck pain and its associated disorders. Spine.

[2] Yan Y, Huang J, Li F, Hu L (2018). Investigation of the effect of neck muscle active force on whiplash injury of the cervical spine. Appl Bionics Biomech.

[3] Yao HD, Svensson MY, Nilsson HJ (2018). Deformation of dorsal root ganglion due to pressure transients of venous blood and cerebrospinal fluid in the cervical vertebral canal. Biomech.

[4] Vasavada AN, Brault JR, Siegmund GP (2007). Musculotendon and fascicle strains in anterior and posterior neck muscles during whiplash injury. Spine.

[5] Carroll L, Holm L, Hogg-Johnson S, Côté P, Cassidy D, Haldeman S (2008). Course and prognostic factors for neck pain in whiplash-associated disorders (WAD). Results of the bone and joint decade 2000–2010 task force on neck pain and its associated disorders. Spine.

[6] Leth-Petersen S, Rotger GP (2009). Long-term labour-market performance of whiplash claimants. J Health Econ.

[7] Peterson G, Nilsson D, Peterson S, Dedering Å, Trygg J, Wallman T, Peolsson A (2016). Changes in dorsal neck muscle function in individuals with chronic whiplash-associated disorders: a real-time ultrasound case-control study. Ultrasound Med Biol.

[8] Rebbeck T (2017). The role of exercise and patient education in the noninvasive management of whiplash. J Orthop Sports Phys Ther.

[9] Brault JR, Siegmund GP, Wheeler JB (2000). Cervical muscle response during whiplash: evidence of a lengthening muscle contraction. Clin Biomech.

[10] Karlsson A, Dahlqvist Leinhard O, Åslund U, West J, Romu T, Smedby Ö (2016). An investigation of fat infiltration of the multifidus muscle in patients with severe neck symptoms associated with chronic whiplash-associated disorders. JOSPT.

[11] Lord SM, Barnsley L, Wallis BJ, Bogduk N (1996). Chronic cervical zygapophysial joint pain after whiplash. A placebo-controlled prevalence study. Spine.

[12] Peterson G, Nilsson D, Trygg J, Peolsson A (2018). Neck-specific exercise improves impaired interactions between ventral neck muscles in chronic whiplash: a randomized controlled ultrasound study. Sci Rep.

[13] Panjabi MM (1992). The stabilizing system of the spine. Part 1. Function, dysfunction, adaptation, and enhancement. J Spinal Disord.

[14] Falla D, Bilenkij G, Jull G (2004). Patients with chronic neck pain demonstrate altered patterns of muscle activation during performance of a functional upper limb task. Spine.

[15] Jull GA, Falla D, Vicenzino B, Hodges PW (2009). The effect of therapeutic exercise on activation of the deep cervical flexor muscles in people with chronic neck pain. Man Ther.

[16] Landén Ludvigsson M, Peterson G, Dedering Å, Peolsson A (2016). One- and two-year follow-up of a randomized trial of neck-specific exercise with or without a behavioural approach compared with prescription of physical activity in chronic whiplash. J Rehabil Med.

[17] Landén Ludvigsson M, Peolsson A, Peterson G, Dedering Å, Johansson G, Bernfort L (2017). Cost-effectiveness of neck-specific exercise with or without a behavioral approach versus physical activity prescription in the treatment of chronic whiplash-associated disorders: analyses of a randomized clinical trial. Medicine.

[18] Overmeer T, Peterson G, Landén Ludvigsson M, Peolsson A (2016). The effect of neck-specific exercise with or without a behavioral approach on psychological factors in chronic whiplash-associated disorders: a randomized controlled trial with a 2-year follow-up. Medicine.

[19] De Pauw R, Coppieters I, Kregel J, De Meulemeester K, Danneels L, Cagnie B (2016). Does muscle morphology change in chronic neck pain patients? – A systematic review. Man Ther.

[20] Elliott JM, O’Leary S, Sterling M, Hendrikz J, Pedler A, Jull G (2010). Magnetic resonance imaging findings of fatty infiltrate in the cervical flexors in chronic whiplash. Spine.

[21] Farrell SF, Smith AD, Hancock MJ, Webb AL, Sterling M (2019). Cervical spine findings on MRI in people with neck pain compared with pain-free controls: a systematic review and meta-analysis. J Magn Reson Imaging.

[22] Smith AC, Albin SR, Abbott R, Crawford RJ, Hoggarth MA, Wasielewski M (2020). Confirming the geography of fatty infiltration in the deep cervical extensor muscles in whiplash recovery. Sci Rep.

[23] Van Looveren E, Cagnie B, Coppieters I, Meeus M, De Pauw R (2021). Changes in muscle morphology in female chronic neck pain patients using magnetic resonance imaging. Spine.

[24] Verhagen AP, Scholten-Peeters GGM, van Wijngaarden S, de Bi R, Bierma-Zeinstra SMA (2007). Conservative treatments for whiplash. Cochrane Database of Systematic Reviews..

[25] Elliott JM, Kerry R, Flynn T, Parrish TB (2013). Content not quantity is a better measure of muscle degeneration in whiplash. Man Ther.

[26] Abbott R, Peolsson A, West J, Elliott JM, Åslund U, Karlsson A (2018). The qualitative grading of muscle fat infiltration in whiplash using fat and water magnetic resonance imaging. Spine J.

[27] Elliott JM, Smith AC, Hoggarth MA, Albin SR, Weber KA, Haager M (2020). Muscle fat infiltration following whiplash: a computed tomography and magnetic resonance imaging comparison. PLoS ONE.

[28] Karlsson A, Peolsson A, Elliott J, Romu T, Ljunggren H, Borga M (2019). The relation between local and distal muscle fat infiltration in chronic whiplash using magnetic resonance imaging. PLoS ONE.

[29] Cagnie B, Barbe T, Vandemaele P, Achten E, Cambier D, Danneels L (2009). MRI analysis of muscle/fat index of the superficial and deep neck muscles in an asymptomatic cohort. Eur Spine J.

[30] Uthaikhup S, Assapun J, Kothan S, Watcharasaksilp K, Elliott JM (2017). Structural changes of the cervical muscles in elder women with cervicogenic headache. Musculoskel Sci Pract.

[31] Ulbrich EJ, Anderson SE, Busato A, Abderhalden S, Boesch C, Zimmermann H (2011). Cervical muscle area measurements in acute whiplash patients and controls. J Magn Reson Imaging.

[32] Vernon H, Mior S (1991). The neck disability index: a study of reliability and validity. J Manipulative Physiol Ther.

[33] Spitzer WO, Skovron ML, Salmi LR, Cassidy JD, Duranceau J, Suissa S (1995). Scientific monograph of the Quebec task force on whiplash-associated disorders: redefining “whiplash” and its management. Spine.

[34] Ludvigsson ML, Peterson G, Peolsson A (2020). Neck-specific exercise for radiating pain and neurological deficits in chronic whiplash, a 1-year follow-up of a randomised clinical trial. Sci Rep.

[35] Peolsson A, Landén Ludvigsson M, Overmeer T, Dedering Å, Bernfort L, Johansson G (2013). Effects of neck-specific exercise with or without a behavioural approach in addition to prescribed physical activity for individuals with chronic whiplash-associated disorders: a prospective randomised study. BMC Musculoskelet Disord.

[36] Hedenstierna S, Halldin P, Siegmund GP (2009). Neck muscle load distribution in lateral, frontal, and rear-end impacts: a three-dimensional finite element analysis. Spine.

[37] Sacher N, Frayne RJ, Dickey JP (2012). Investigating cervical muscle response and head kinematics during right, left, frontal and rear-seated perturbations. Traffic Inj Prev.

[38] Giroud M, Karbiener M, Pisani DF, Ghandour RA, Beranger GE, Niemi T (2016). Let-7i-5p represses brite adipocyte function in mice and humans. Sci Rep.

[39] Elliott JM, Rueckeis CA, Pan Y, Parrish TB, Walton DM, Linnstaedt SD (2021). MicroRNA let-7i-5p mediates the relationship between muscle fat infiltration and neck pain disability following motor vehicle collision: a preliminary study. Sci Rep.

[40] Smith AC, Parrish TB, Hoggarth MA, McPherson JG, Tysseling VM, Wasielewski M (2015). Potential associations between chronic whiplash and incomplete spinal cord injury. Spinal Cord Ser Cases.

[41] Owers DS, Perriman DM, Smith PN, Neeman T, Webb AL (2018). Evidence for cervical muscle morphometric changes on magnetic resonance images after whiplash: a systematic review and meta-analysis. Injury.

[42] Cagnie B, Dolphens M, Peters I, Achten E, Cambier D, Danneels L (2010). Use of muscle functional magnetic resonance imaging to compare cervical flexor activity between patients with whiplash-associated disorders and people who are healthy. Phys Ther.

[43] Elliott J, Jull G, Noteboom JT, Darnell R, Galloway G, Gibbon WW (2006). Fatty infiltration in the cervical extensor muscles in persistent whiplash-associated disorders: a magnetic resonance imaging analysis. Spine.

[44] Uhlig Y, Weber BR, Grob D, Müntener M (1995). Fiber composition and fiber transformations in neck muscles of patients with dysfunction of the cervical spine. J Orthop Res.

[45] Weber KA, Smith AC, Wasielewski M, Eghtesad K, Upadhyayula PA, Wintermark M (2019). Deep learning convolutional neural networks for the automatic quantification of muscle fat infiltration following whiplash injury. Sci Rep.

[46] Weber KA, Abbott R, Bojilov V, Smith AC, Wasielewski M, Hastie TJ (2021). Multi-muscle deep learning segmentation to automate the quantification of muscle fat infiltration in cervical spine conditions. Sci Rep.

